# Current controlled vocabularies are insufficient to uniquely map molecular entities to mass spectrometry signal

**DOI:** 10.1186/1471-2105-16-S7-S2

**Published:** 2015-04-23

**Authors:** Rob Smith, Ryan M Taylor, John T Prince

**Affiliations:** 1 Department of Computer Science, University of Montana, 59812 Missoula, USA; 2 Department of Chemistry, Brigham Young University, 84606 Provo, USA

**Keywords:** Controlled Vocabulary, Proteomics, Lipidomics, Metabolomics

## Abstract

**Background:**

The comparison of analyte mass spectrometry precursor (MS1) signal is central to many proteomic (and other -omic) workflows. Standard vocabularies for mass spectrometry exist and provide good coverage for most experimental applications yet are insufficient for concise and unambiguous description of data concepts spanning the range of signal provenance from a molecular perspective (e.g. from charged peptides down to fine isotopes). Without a standard unambiguous nomenclature, literature searches, algorithm reproducibility and algorithm evaluation for MS-omics data processing are nearly impossible.

**Results:**

We show how terms from current official ontologies are too vague or ambiguous to explicitly map molecular entities to MS signals and we illustrate the inconsistency and ambiguity of current colloquially used terms. We also propose a set of terms for MS1 signal that uniquely, succinctly and intuitively describe data concepts spanning the range of signal provenance from full molecule downs to fine isotopes. We suggest that additional community discussion of these terms should precede any further standardization efforts. We propose a novel nomenclature that spans the range of the required granularity to describe MS data processing from the perspective of the molecular provenance of the MS signal.

**Conclusions:**

The proposed nomenclature provides a chain of succinct and unique terms spanning the signal created by a charged molecule down through each of its constituent subsignals. We suggest that additional community discussion of these terms should precede any further standardization efforts.

## Background

Liquid-chromatography mass spectrometry (LC-MS) is a ubiquitous platform for proteomic (and other "omic") investigations [1]. MS signal from hundreds to millions of ions can be quantitatively compared across experimental conditions in a fairly robust and repeatable way [2]. Analyte quantities are captured directly in MS signal (aka MS1), while analyte identities are often elucidated or confirmed using MS/MS (aka MS2) fragmentation spectra [2].

Confidently matching MS1 analyte signal between runs ("correspondence") is difficult with complex samples [3], so a variety of approaches to circumvent this problem have been explored. Multiple reaction monitoring (MRM) can be effective for monitoring a relatively small number of pre-selected analytes with a high degree of confidence, but it is unsuited to discovery-based experiments. MS/MS based approaches (e.g. iTRAQ [4] and spectral counting [5]) are also popular alternatives. However, due to low MS/MS capture rates (10-20%) and true positive database match rates (<60%) [2], MS/MS driven approaches lack sensitivity compared to MS1-based approaches. Although a data independent acquisition (DIA) approach may address some of the sensitivity deficiencies of MS/MS for identification, DIA does not of itself address difficulties in correspondence and quantitation. Hence, despite the availability of alternative approaches, the ability to match MS1 signal across experimental conditions is still highly desired.

Numerous efforts, large and small, have focused on using MS1 signal to compare analyte quantities. Ideally, solutions would focus on one of the several complex individual steps for data processing [6]. However, most are released as end-to-end solutions (e.g., SuperHirn [7], MaxQuant [8], XCMS [9], and Skyline [10]). This makes comparison to other competing algorithms virtually impossible, and is at least partially responsible for the lack of critical evaluations in the literature [11], since testing a subcomponent of a full software system requires re-implementing that portion of the algorithmic pipeline. Our awareness of this problem has been accentuated as we have recently undertaken a survey in each of several of the modular subproblems of LC-MS quantification. When one must distinguish the algorithmic details of several methods, or worse, implement them in code, one becomes painfully aware of the ambiguity in the terms currently used in MS data processing descriptions.

The lack of standard terminology has stagnated LC-MS data processing progress. Without consistent, clear terminology researchers have no handles for searching the literature, requiring onerous literature searches which fail to capture all relevant publications. Besides lack of access to possibly improved results, this leads to massive duplication of effort and few cross-tool evaluations since researchers are unaware of related efforts. A well defined vocabulary and problem domain also encourage and aid new-comers to the field--which currently poses a significant learning curve [12]--improving solutions to difficult data processing challenges. It is also much easier to re-implement solutions when both the *what *and *how *of a process are clearly understood. Hence, an increase in term clarity has immediate impact on reproducibility--a requirement firmly enforced for sample preparation and wet-lab processing protocols but which is almost completely unenforced for data processing descriptions [11]. The rigor and statistical biases of MS data processing algorithms--which usually must be evaluated qualitatively by the user due to a lack of quantitative comparison [11]--are likely to be miscommunicated in the literature without a clear nomenclature, leading to overstating the significance of and confidence in experimental results.

HUPO-PSI [13] and IUPAC [14] have presented controlled vocabularies (CVs) for mass spectrometry. However, the shotgun-pattern coverage of current CVs falls short of a systematic coverage of the full range of relevant data processing concepts. In the literature, these gaps are filled with arbitrary and/or overloaded ambiguous terms, precluding experimental reproducibly and comprehension. To motivate the need for precise terminology in hand, we begin by showing how terms from current official ontologies are too vague to explicitly map molecular entities to MS signals, and we illustrate the inconsistency and ambiguity of current colloquially used terms. The details of what terms the PSI and IUPAC committees choose to fill the present need will be a long process. As a critical first step towards eventual standardization, we have crafted a systematic and consistent map of terms that span the range of the required granularity to describe MS data processing. By approaching the nomenclature task from the perspective of the molecular provenance of the MS signal, the proposed nomenclature provides a chain of succinct and unique terms allowing unique description of every data concept from the signal created by a charged molecule down through each of its constituent subsignals.

That a community standard controlled vocabulary is necessary is abundantly obvious from the literature. Consider, for instance, the usage of two of the most common MS-omics data concept labels. These lists are by no means exhaustive in references or instances.

The term *feature *is used for:

• The whole 3-d signal produced by one molecule type (such as a peptide), across one or more charge states [15-17].

• Any of the many possible summaries of that signal as a single 3-tuple (m/z, RT, intensity) [7,18,19].

• Any subsignal of that whole 3-d signal in m/z, RT, or intensity [2].

The term *peak *is used for:

• The whole 3-d signal produced by one molecule type (such as a peptide), across one or more charge states [20,2,16].

• Any of the many possible summaries of that signal as a single 3-tuple (m/z, RT, intensity) [16,19,20,2].

• Any subsignal of that whole 3-d signal in m/z, RT, or intensity [16,15,21,8-25].

In both cases, although each publication makes a distinction between the two terms, the difference is lost across the literature. These lists could be much longer if it was possible to identify the specific subsignal referred to by the author, a task impossible with the generality of current terms.

It should be abundantly clear that these terms convey very little useful information--certainly insufficient information for reproducibility. Even terms with consistent use, there is a general lack of scope. For example, evenly the seemingly descriptive term *monoisotopic peak *cannot convey exactly what level of data processing has been used on the signal. Is it the 2-d signal of the most abundant isotope signal in the whole 3-d signal produced by one molecule type [15], the integration of that signal [25], the integration of the intensity of the whole 3-d signal into one 3-tuple [26,16], or something else? All of these uses fit the commonly understood definition, but none are specific enough to readily discern from just the term.

These examples briefly illustrate the ubiquity of overloading (using one term to mean more than one concept). Overloading treats a term as a variable, whose meaning must be defined in detail for the scope of each publication it appears in. An adequate definition takes significant thought, some descriptive text, and usually a descriptive image. There simply isn't ample space in each manuscript to define a custom set of terms for MS-omics data processing. This results in insufficient definitions for terms or no definitions at all.

For instance, in a published algorithmic review paper [22], the author provides a definition list to allow for the use of mathematical algorithm descriptions. These include symbols for *peak *area, number of *chromatograms*, *peak *maximum, *peak *end, *peaks *detected in a mass channel, raw height of a *peak*, and *peaks *detected in a *chromatogram*. But what is a *peak *? What is a *chromatogram*? As illustrated, these terms are not universally defined, and the author does not define them. Subsequently, the algorithms in the paper are irreproducible unless the reader is able to correctly guess the definition of these terms intended by the author, as the result is dependent upon the term definitions.

Reproducibility is, in fact, at the heart of the nomenclature problem. An algorithm description is rendered useless if the terms used within it are ambiguous or undefined. In a modular approach to pipeline algorithm creation and testing [6], data processing methods prior to the pipeline module of interest have to be exactly describable with concise terms to assure properly formatted input. In evaluating algorithms, knowing the exact format of the input data informs interpretation of the algorithm. If the data format is known, as well as the process used to transform, segment, and/or reduce the original, one can know what biases are intrinsic in the algorithm, as well as immediately suggest improvements. For example, an algorithm that uses the whole 3-d signal from one charge state of a molecule has more information to distinguish differences in a correspondence task than one that has reduced that signal to a single 3-tuple representation. Do current CVs capture the required degree of descriptive specificity?

### Why current CV terms are insufficient

IUPAC [14] and HUPO-PSI [13] are organizations that specialize in standardizing nomenclature. Their significant and useful controlled vocabularies address all aspects of MS experimentation. To date, most of the thousands of terms in these overlapping controlled vocabularies are focused on wet lab protocol and instrumentation. Although there are some terms relevant to data processing, the need for a systematic and consistent approach with more coverage is apparent.

#### Current CV MS data processing terms are ambiguous and inconsistent

**Figure 1:**
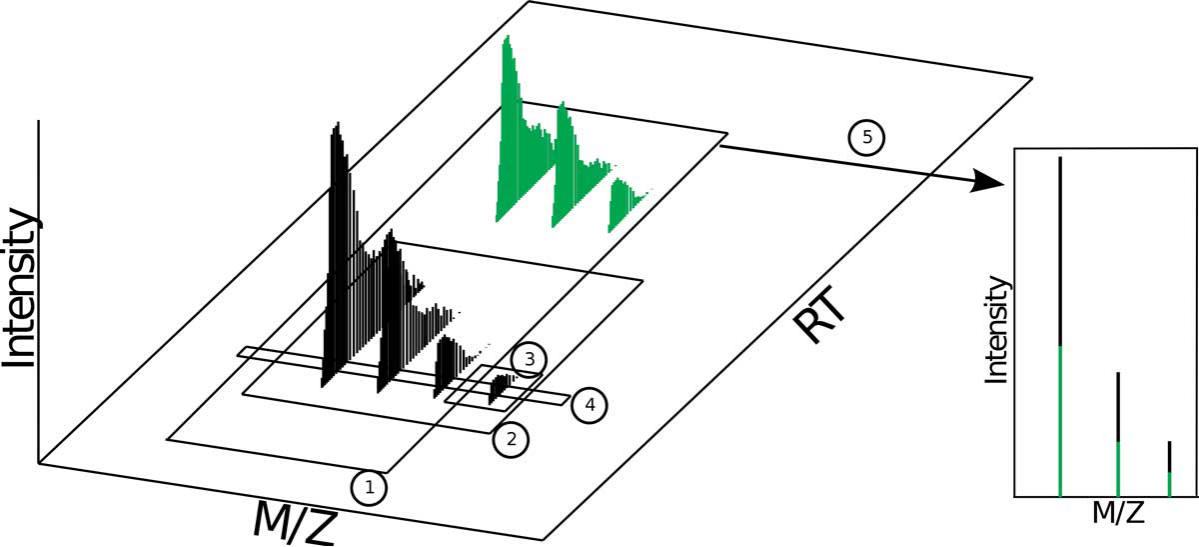
**Any of the boxed signal in this figure can correctly be called a *chromatogram*, a *mass spectrum*, or a *profile spectrum *as defined by existing CVs**. Additionally, the IUPAC terms *isotope pattern *and *isotope cluster *can refer to the signal in box 1, 2, or 3. It is clear that these terms pertain to too many distinct concepts to be clear. Note that existing nomenclatures have no way of distinguishing between these distinct data concepts, which can include parts of one or more combined signals.

The HUPO-PSI-MS OBO has more MS data processing terms than IUPAC. Most are generic to the point of extreme ambiguity. For example, the term *mass spectrum *refers to any segment of data with m/z and abundance axes: "a plot of the relative abundance of a beam or other collection of ions as a function of the mass-to-charge ratio (m/z)." This could refer to a host of different data segmentations, and seems to be a synonym for another term, *profile spectrum*, defined as "A profile mass spectrum is created when data is recorded with ion current (counts per second) on one axis and mass/charge ratio on another axis" (see Figure 1).

An equally ambiguous complementary term is provided to refer to the time and abundance axes: *chromatogram*, defined as "the representation of detector response versus time." This definition is not scoped at all with respect to the molecular entities whose signals are being measured. The term applies equally at any scope. In other words, any plotted entity that shows RT vs intensity qualifies as a chromatogram. Likewise, the term *total ion current chromatogram*, defined as the "chromatogram obtained by plotting the total ion current detected in each of a series of mass spectra recorded as a function of retention time" fails to imply any sort of scoping, and, worse, can correctly apply to any entity that qualifies as a *chromatogram*.

**Figure 2:**
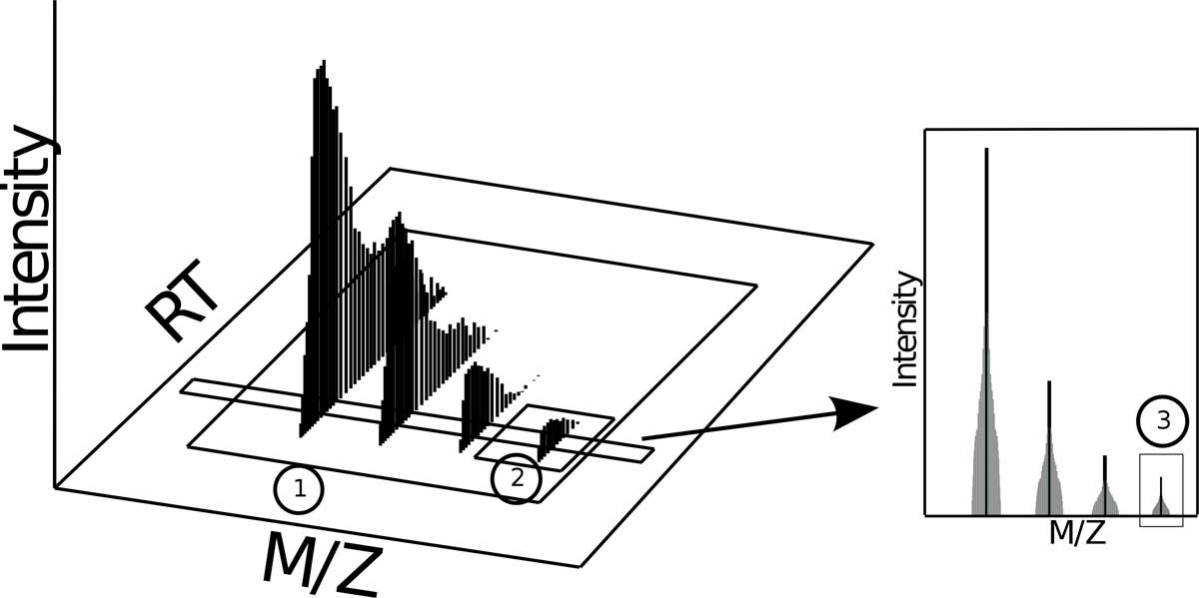
**The PSI CV states that a *peak *is "A localized region of relatively large ion signal in a mass spectrum**." Note that each of the numbered data segmentations in this figure qualify as a *peak *according to the PSI CV. In the literature, each of the illustrated concepts can also be found referred to as a *feature*.

The term *peak *is defined in the PSI CV as "a localized region of relatively large ion signal in a mass spectrum." As defined this term cannot discriminate between a host of distinct data concepts (see Figure 2). Among other associations, the term *peak *is used as a qualifier to describe a process officially named *peak picking*, when profile data is converted to centroid data. Thus, a *peak *can be any signal region (large or small) that consists of one or more centroids, which means any size subset of any data in any projection could be called a *peak*. The term has absolutely no specificity. The term *area peak picking *has a very unclear definition: "spectral peak processing conducted on the acquired data to convert profile data to centroided data. The area defined by all raw data points that belong to the peak is reported." Intuitively, one would assume that *area peak picking *has to have a different meaning than regular *peak picking*, yet the distinction is not evident from the definitions, given the only difference is the addition of "The area defined by all raw points that belong to the peak is reported." What is meant by "peak"? Reported to/by what? Examples such as this suggest that at least some of the current CVs came about through submission of terms from software user manuals rather than a curated, concentrated effort.

It is unclear what a *centroid spectrum *is. The definition states "processing of profile data to produce spectra that contains discrete peaks of zero width. Often used to reduce the size of dataset." However, a spectrum, as defined, can only have two dimensions: m/z and intensity. Since a *spectrum *cannot have an RT dimension, a *centroid spectrum *must be the same thing as a *peak*. Do they instead mean a *peak picked *profile signal summed through RT? It is unclear.

**Figure 3:**
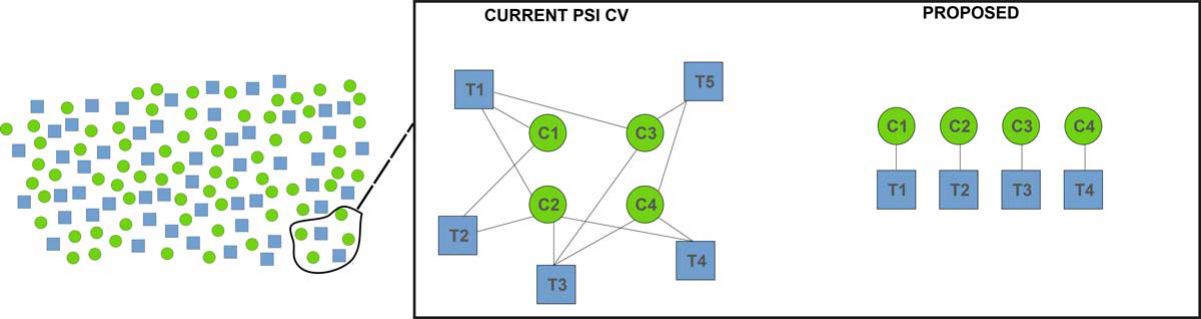
**The PSI controlled vocabulary has over 2,400 entries**. Only ten or so of these entries provide data processing concepts related to MS processing. Far from trying to compete with PSI or IUPAC, which are full-purpose controlled vocabularies, our proposal in this manuscript is to question the small subset of terms they provide for utility in describing MS processing. We propose a set of terms for MS1 data processing constructed of a limited number of base and qualifier terms that allow a vast number of MS1 data concepts to be succinctly, precisely, and intuitively described. The current ambiguous nomenclature yields situations where a given term refers to more than one concept and/or distinct terms refer to the same concept. An unambiguous nomenclature is composed of terms which refer to only one concept each, and no two terms refer to the same concept.

Finally, the inconsistency of the PSI CV is apparent. For example, the term *feature *is used in at least 25 definitions, but it is never defined. It is used to refer to at least a few different concepts, including the idea of a program parameter (MS:1000498, MS:1001760, MS:1002426), the isotopic envelope (MS:1001826, MS:1001827, MS:1002163), a PSI-CV *mass spectrum *(MS:1002166, MS:1002167, MS:1002168), and probably others (probably because it is unclear what is being described in the other 16 definitions). Another term, *mass trace*, is similarly used in several definitions yet never defined. The implied use, like *feature*, overlaps the definitions of *peak *and *chromatogram*, making for considerable ambiguity (see Figure 3). In addition to these generic terms, the PSI CV provides two specific data concepts: *deisotoping *and *charge deconvolution*. *Deisotoping *is referred to as "the removal of isotope peaks to represent the fragment ion as one data point and is commonly done to reduce complexity. It is done in conjunction with the charge state deconvolution." The concept described is worthy of a definition, but the one provided can be improved upon. A fragment ion is not a data signal, but a molecular object. However, deisotoping is an operation on a data signal. Additionally, this term should not be specific to MSn fragment ions, but also applies to non-fragmented MS1 data, such as an MS1 *isotopic envelope*. Our nomenclature expands this term to include the logical wider use. *Charge deconvolution *is defined as "the determination of the mass of an ion based on the mass spectral peaks that represent multiple-charge ions." *Deconvolution *is already a widely used signal processing term (also used in MS processing e.g. in [27]) for resolving two overlapping signals into their constituent parts). The PSI definition redefines an already widely used term to mean something other than what it means in all other contexts. What's more, the definition focuses on mass determination, not signal manipulation. It should be replaced.

#### Current CVs don't describe all necessary data concepts for MS data processing

Crucial concepts for MS data processing are missing from the current CVs. Most of these relate to more specific concepts at higher granularity than is currently offered by the PSI and IUPAC CVs. A rigorous CV should allow for unique terms to describe signals from a peptide/lipid/metabolite down to individual data points. With the current nomenclatures, it is impossible to describe data processing algorithms' details using standard terms.

Explicit mapping of molecular entities to the signal their detected ions produce is essential to achieve clarity at all scopes of granularity. The PSI and IUPAC terms do not quite do this. For example, the best term to refer to a host of loosely related concepts is *ion*: "an atomic, molecular, or radical species with a non-zero net electric charge." Ion is a proper and correct term that is general to science, and this is the widely used definition. However, this term applies to a charged item of any size, and cannot distinguish among the instances of interest (e.g. proteins in a proteomics experiment, lipids in a lipidomics experiment) and the smaller molecular charged units that are detected in a mass spectrometer experiment. Specific and unique terms would provide the needed descriptive granularity.

#### An incomplete CV impedes algorithm implementation/comparison

Writing code is a mathematically precise activity. Reproducibility requires the exact same equation, as it were, to be reproduced. Reproducibility requires specificity and clarity. Any ambiguity, overloading, or lack of detail makes the process impossible. Frequently, it becomes necessary to code up a published algorithm. This could be because the algorithm was published independent of a software platform, or because you want to see the results of one particular module of a full-service program. As a case study, we recently attempted to code up one module of the MaxQuant algorithm [8] in order to compare results of a feature detection algorithm. The following text is the pertinent portion, and demonstrates just how difficult it can be to parse through an algorithmic description without a good nomenclature, even in a well-written, top-tier-published manuscript:

...peaks are detected in a conventional two-dimensional (2D) way by first searching for local maxima of the intensity as a function of m/z. The lower and upper limits of the m/z interval for a 2D peak...are then determined by moving from the maximum position to smaller and larger m/z values, until either the intensity has dropped to zero, or a local intensity minimum has been reached....The centroid position of a 2D peak is then determined...If the peak consists of only one raw data point, then the m/z value of that point is taken as the centroid position. If there are two raw data points in a peak, then the centroid position is defined as the average of the two raw m/z values, weighted by the raw intensities....the 2D peaks in adjacent MS spectra are assembled into 3D peak hills over the m/z-retention time plane. Two peaks in neighboring scans are connected whenever their centroid m/z positions differ by less than 7 ppm. If for a given centroid in MS scan n no matching centroid is found in scan (n+1) in the ±7 ppm mass window, then it is checked if there is a centroid in scan (n+2) in the same mass window to continue the peak in time. We adjusted the window size to 7 ppm by visual inspection of many very low abundant peaks....A 3D peak is defined as the maximal chain of 2D peaks that results from connecting the centroids in time direction in the described way. At least two centroids have to be matched together to form a 3D peak, i.e. centroids that cannot be matched to centroids in the two previous or the two next scans are discarded....If a minimum is found whose value is 1/1.3 of the lower of the two local maxima the 3D peak is split into two at the minimum position...

With unclear terms, translating a manuscript into code is very difficult, and very unlikely to produce what the author intended.

Ambiguity also affects practitioners who do not write code. While programmers could invest the required time to study the source code to fill in details missing from the published algorithmic description, practitioners are limited to published descriptions. In the process of writing an exhaustive LC-MS correspondence survey [3], we recently parsed through over 50 manuscripts describing correspondence algorithms, attempting to describe distinctions between them. Each manuscript had its own unique ill-defined vocabulary. It was very difficult to decipher algorithmic differences from the manuscript alone, even with much more time and effort than would be available to a standard user.

#### An incomplete CV impedes literature reviews

Ambiguity also leads to publication of non-novel approaches due to a general unawareness of algorithmic detail, a phenomenon we noted in the above-mentioned correspondence review. This is a compounding problem, as it creates even more methods that must be compared against and even more papers to read. For example, there is probably an algorithmic difference between TracMass2 [28] and the corresponding module of MaxQuant. However, neither algorithm is described specifically enough for the reader or reviewers to tell, as witnessed by the lack of a citation to MaxQuant. This is just one case of the ubiquitous unintentional plagiarism in the MS data processing literature. Clear nomenclature makes a thorough literature search tractable, alleviating the burden of discerning differences in algorithms and minimizing the republication of non-novel approaches.

#### Community awareness and an immediate, intermediate response is the correct way of addressing this need

All controlled vocabularies are works in progress. Standards committees are best at crystallizing and refining accepted practice, but the onus to invent or select appropriate terminology lies foremost with the community itself. A good example of this was the creation of a standard spectrum exchange format. The mzXML format was created and published by a small group of researchers [29]. After several years of use the HUPO PSI mass spectrometry working group produced the mzML format which was able to draw upon the experience gained from use of the mzXML format. Although a data format and not a CV, the success of mzML shows the good that can come of a manuscriptdriven approach. mzXML was originally published as a manuscript, the sole product of a small group of researchers who noticed a problem and forwarded a solution. This was the genesis of community traction that culminated in the mzML standard, a significant step forward for all mass spectrometry users. An official nomenclature culminates with IUPAC [14] and HUPO-PSI [13] standards but the community cannot realistically expect nomenclature to begin there.

At present, the controlled vocabularies simply do not have coverage in the terms related to data processing. Because the problem is so extensive, and because opinions run strong in the domain of nomenclatures, this problem is best solved by drawing attention to shortcomings while providing a framework for unambiguous terms. A manuscript to draw attention to areas that can be improved are a viable means of correcting them, as demonstrated in other CVs [30]. As we have shown by enumerating collisions, inconsistencies, and gaps in current terms, no small group of experts can successfully bring about a CV independent of an active, involved community, particularly when data processing represents only a small subset of the larger experimental community. It is unfair to represent the data processing portion of the current PSI CV as the calculated and careful end product of a long, focused deliberation. Inspection of the terms makes it clear that this is not only a living, changing document, but (at least in terms of the data processing terms) seems to be, at least in part, an uncoordinated amalgamation of terms from different software groups. We submit that fostering community discussion in a peer-reviewed venue is at least as valid as open, uncoordinated, and seemingly minimally curated submissions to a standard.

## Results and discussion

**Figure 4:**
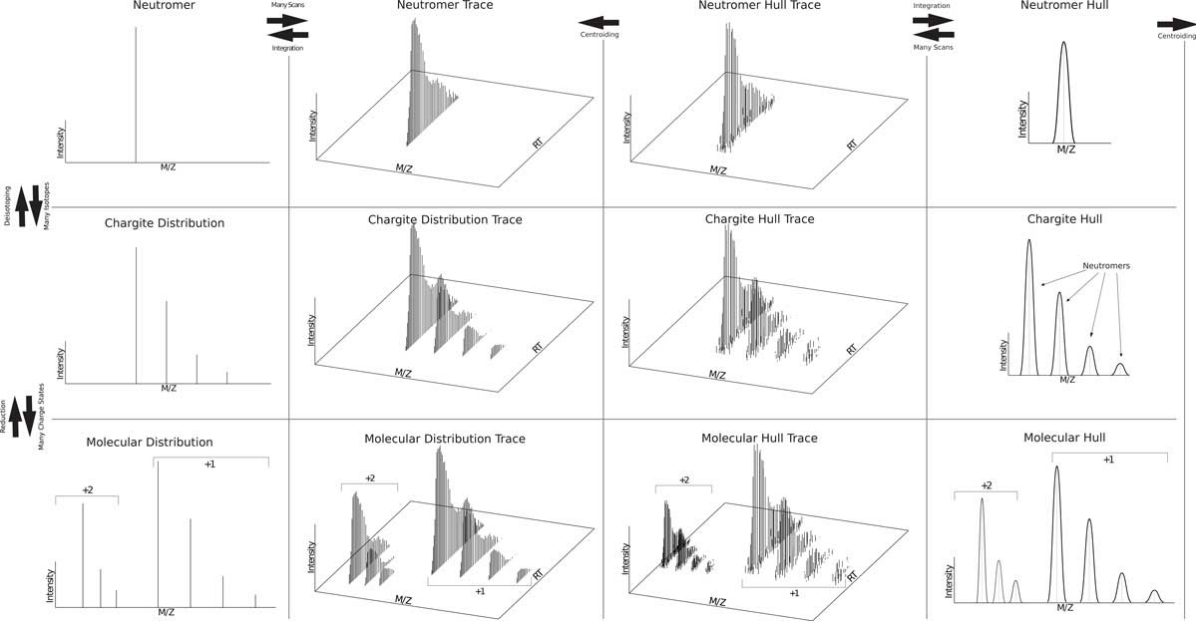
**The proposed nomenclature provides descriptive handles across dimensions and across granular scope while simultaneously providing a mechanism to disambiguate data reduction products (see Proposed Terms)**.

The PSI CV data processing terms are scant, inconsistent, and ambiguous for describing MS data processing. We propose a system for generating terms that allows greater unambiguous coverage of currently addressed concepts in a consistent and intuitive manner, facilitating reproducibility, comprehension, and searchability of data processing algorithms. The motivation at the heart of our proposed nomenclature is to explicitly map causal molecular entities to the signals they produce. An overview of all terms is presented in Figure 4.

### Base terms

*Molecule *- The composite signals across charges of a unit that accepts charge. For instance, a lipid in a lipidomics experiment or a peptide in a bottom-up proteomics experiment (see Figure 4).

*Chargite *- A unit that accepts charge at a particular charge state (see Figure 4).

*Neutromer *- A *neutromer *is the portion of a *chargite *with a common number of neutrons (see Figure 4; no distinction is made in this context among molecules where the neutron is associated with different atoms or kinds of atoms).

**Figure 5:**
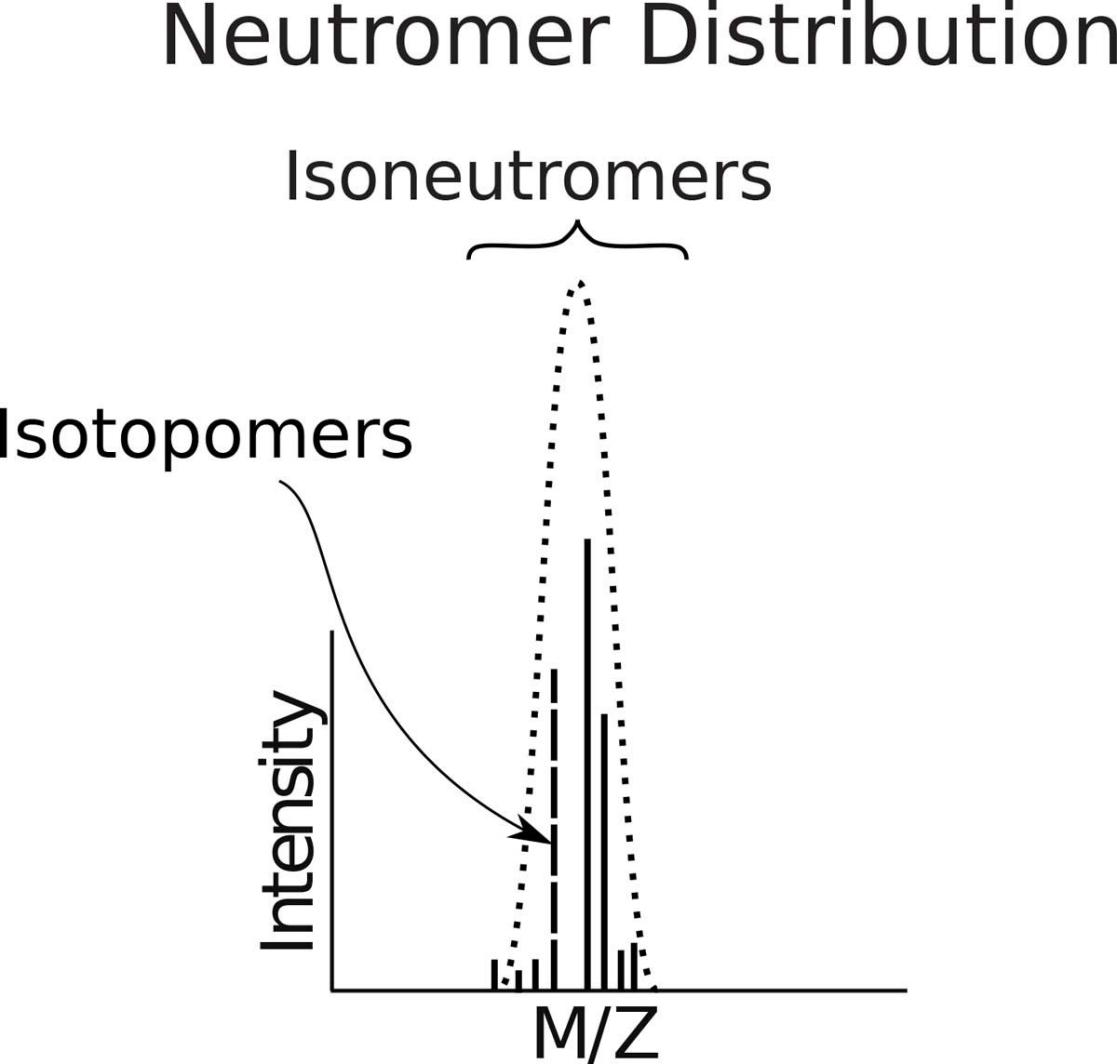
**A *neutromer distribution *consists of *isoneutromers*--isomers that differ only in the number of neutrons they possess**. They comprise a *neutromer hull *(dotted line). Each *isoneutromer *is in turn comprised of the set of *isotopomers*, those *neutromers *with identical isotopic composition but different position.

**Figure 6:**
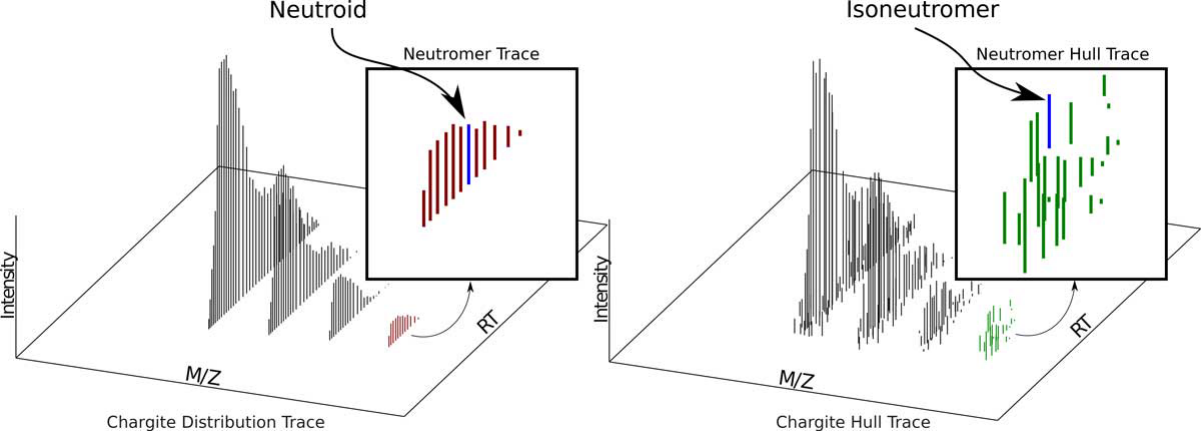
**The proposed nomenclature provides a distinctive term for each m/z, RT, intensity tuple in the mzML file**.

*Isoneutromer *- Isomers that differ only in the number of neutrons they possess (see Figures 5 and 6).

*Isotopomer *-Isomers with identical isotopic composition but different structure (see Figure 5).

*Neutroid *- An *instantaneous centroided neutromer hull *(see Figure 6).

### RT dimensional terms

These terms provide us with specific descriptive ability for the RT dimensional component of the data concept, allowing us to distinguish between signals that continue through the RT dimension and signals that occupy only one RT.

*Trace *- A *trace *indicates a signal that extends into the RT dimension (see columns 2 and 3 of Figure 4). For example, when we combine *chargite *and *trace*, we get a *chargite trace*, which is the unique whole (meaning throughout RT) accumulated (meaning throughout a run) signal generated by one *molecule */ charge state combination consisting of one or more *neutromer traces *equally spaced m/z 1/z apart (see Figure 4). This can (and should) be further qualified as either a *chargite distribution trace *or a *chargite distribution hull*. Likewise, a *neutromer hull *is the unique whole (meaning throughout RT) signal generated by the accumulation of instances of a given *molecule *at a given charge state whose molecular formula contains the same isotopic composition (see Figure 4). A *neutromer trace *is the same signal but subjected to a centroiding algorithm reducing it from a collection of *instantaneous neutromer hulls *to a collection of *neutroids *(see Figure 6). A *molecular distribution trace *is the set of whole (meaning throughout RT) *chargite distribution traces *generated by one molecule across multiple charge states (see Figure 4).

*Integrated *- An integrated object has been summed through the RT dimension. For example, if we take a *neutromer trace *and sum its constituent *neutroids*, we will end up with a single 3-tuple consisting of m/z, RT, and intensity that can accurately be called a *neutromer *(see Figure 4). However, by calling it an *integrated neutromer trace*, we retain a unique description of the original data structure as well as the data reduction process used.

*Max *- The qualifier *max *implies that this object is the *instantaneous *slice of a trace object at a the RT of greatest intensity. Max objects look exactly like those with that are *integrated*, however, this qualifier indicates that we are looking at a slice of the data structure in time, not a summation or average of the data through time.

*Average *- The data concepts described by the qualifier *average *are, in appearance, the same as those in *integrated*, however the process to generate them involves taking the average of the intensity of the composite points, not the sum.

*Instantaneous *- The qualifier *instantaneous *implies that this object is a spectral slice of a trace object at a given RT. The *instantaneous *objects look exactly like those with that are *integrated*, however, this qualifier indicates that we are looking at a slice of the data structure at one scan time, not a summation or average of the data through time.

### M/Z terms

When reducing data from its initial state (Figure 4 columns 2 and 3), the resultant data concept (Figure 4 columns 1 or 4) should be amended to include the data processing technique (see proposed terms below). For example, a *chargite distribution trace *can become an *instantaneous chargite distribution *if the data reduction consists of selecting a random scan of the *chargite distribution*. In this way, data reduction products that can result from multiple operations are disambiguated.

*Hull *- Refers to the convex hull fitted over the subsignal data points for the given scope. For example, a *neutromer hull *is the convex hull over the *isoneutromers *for a given *neutromer*.

*Distribution *- Connotes a the collection of *neutroids *from a *chargite *or *molecule *in a particular RT and m/z scope. For example, an *max chargite distribution *refers to the centroided *chargite hull trace *at the apex intensity (see Figure 4).

**Figure 7:**
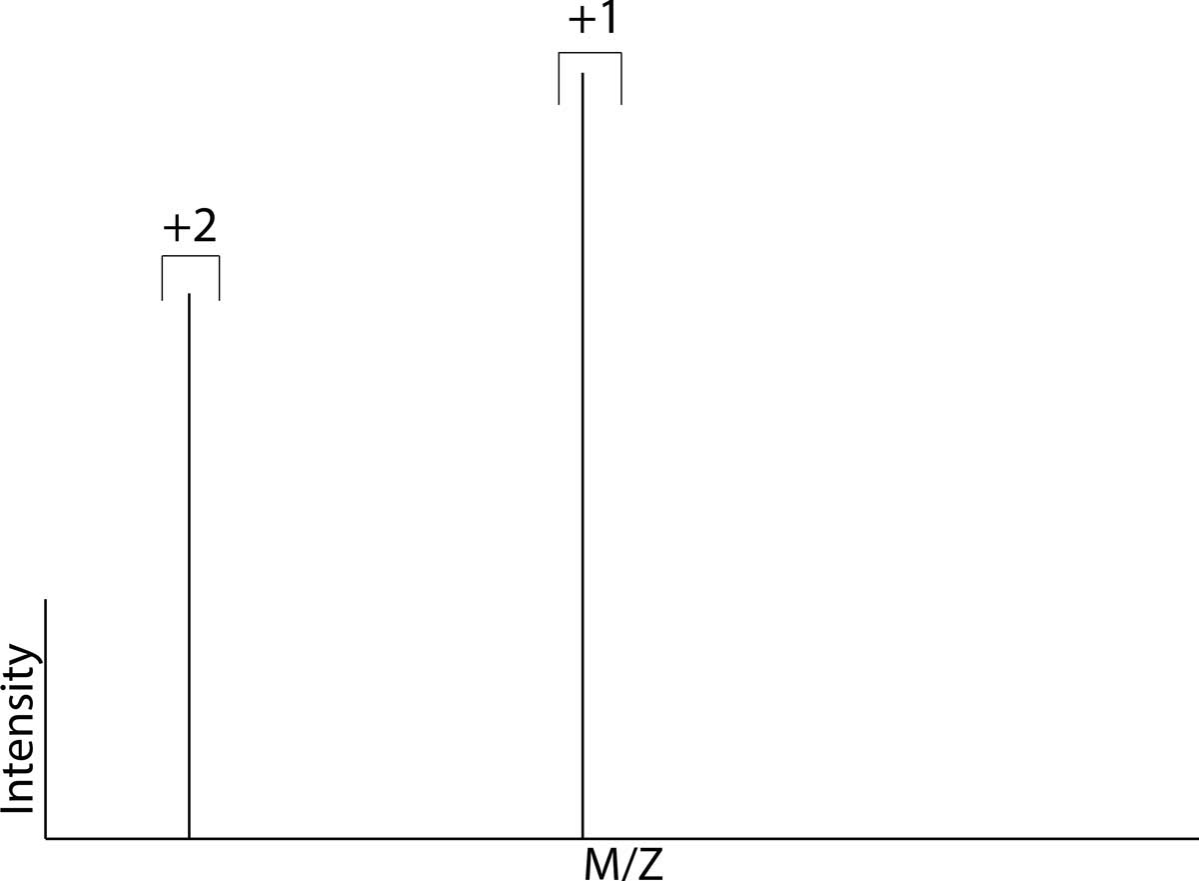
***Deisotoped molecular distribution *- the set of *neutromer centroids *resulting from the composition of all *neutromers *in each *chargite distribution*/*chargite hull *in a *molecular distribution*/*molecular hull *(see Figure 7)**.

*Deisotoped *- The qualifier *deisotoped *implies that a *chargite*'s intensities have been summed through the m/z dimension (see Figure 7). For clarity, *deisotoped *should be used in combination with an RT qualifier indicating the method used for reducing through the RT dimension, such as *integrated*.

*Reduced *- The qualifier *reduced *implies that the object has been combined through reducing charge states to the lowest common charge state. For instance, a *reduced molecular distribution *is the set of the composition of all *chargite distributions *in the *molecular distribution *(see Figure 4).

### Clearer than colloquial terms

Our suggested vocabulary alleviates much of the ambiguity in the current naming schemes employed in the literature. The following examples illustrate how the proposed vocabulary untangles the currently obfuscated terms in use.

*Chargite trace *describes a concept for which the following terms have all been used: an *eluting isotopic distribution *[31], a *chromatogram *[22], an *isotope series *[22], an *isotope pattern *[8], an *isotope-resolved mass spectrum *[32], an *ion series *[33] and an *isotopic cluster *[26,27]. None of these terms differentiate between the concepts we refer to as *chargite trace, instantaneous chargite hull, max chargite distribution*, etc.

*Neutromer traces *have been referred to as *eluting isotopes *[31], *single ion chromatograms *[22], *peaks *[22,21,8], *mass spectra *[24], and *peak hills *[8]. Each of these terms are unclear. The problem with the term *chromatogram *is that is does not specifically refer to the elution profile of a single *isotope*. For example, an *extracted ion chromatogram *is an m/z slice of data that can extend across an entire run's RT. Any term that uses *peak *is bound to be confusing due to the overuse of the term. Like *chromatogram*, a *mass spectrum *can technically stretch across an entire m/z range and therefore does not specifically describe the m/z window of a specific *molecule*.

*Integrated chargite distribution trace *has been called an *isotope pattern *[21]. However, many other concepts can accurately be called *isotope patterns*, such as a *max isotopic envelope *or an *averaged isotopic envelope*.

Using this nomenclature, it is much simpler to clearly and unambiguously describe an MS data processing algorithm. The following text is the translation of the above-quoted MaxQuant text translated into the proposed nomenclature.

Overlapping and/or contiguous *neutromer hull traces *are deconvolved by bisecting all contiguous *neutromer hull traces *with a local maximum bordered by local minima. Each *neutromer hull *is centroided by taking the weighted average of the m/z values of its *isoneutromers*. *Neutromer traces *are constructed from *neutroids *by the following method: For each scan, each *neutroid *within 7 ppm of an isotope trace from the previous scan or penultimate scan is aggregated to the closest (in ppm) *neutromer trace*. All other *neutroids *are considered new *neutromer traces *for future scans. Any *neutromer traces *with only one *neutroid *after all scans are included are culled. A postprocessing mechanism to address erroneously appended *neutromer traces *splits the *neutromer trace *anywhere a *neutroid *is found with intensity less than or equal to 1/1.3 of the lesser intensity of two surrounding local maxima.

Not only does this text more readily reduce to code, it is easier to understand and takes up about half the text of the original. No term is used to mean more than one specific concept. The terms have a one to one mapping to the concept they refer to. This clarity readily expands to algorithmic descriptions across the subproblems of MS data processing, such as LC-MS correspondence.

### Expected objections

Having discussed this nomenclature with many of our colleagues, we anticipate some objections and will address the most common here.

#### This nomenclature competes with PSI/IUPAC

We are not advocating for a replacement of either IUPAC or PSI controlled vocabularies, rather arguing that the subset of terms relevant to data processing have insufficient coverage and are ambiguous where defined (see Figure 3). We are arguing that those terms relevant to data process discussed here ought to be replaced, and those missing ought to be added.

#### Why don't you submit these to PSI?

We are planning on doing so. However, this manuscript is not an attempt to change PSI. Standardizations do not drive the community, the community drives standardizations. There is a current dearth of appropriate terms to describe MS data processing. We have provided solid evidence that this is a problem, and we have proposed a nomenclature that solves that problem. We do not have a stake in PSI and cannot control their nomenclature. However this nomenclature can be used immediately regardless of community response.

#### You have no data format

The community does not recognize a controlled vocabulary and an data format (XML schema) as the same thing, as demonstrated by the fact that PSI has data formats and a CV, and they are separate products. The first step is establishing terms that describe these concepts. Producing a data format that represents these data concepts is a different problem that will have to be addressed in the future. Having a data format before establishing that this is a problem, let alone before coalescing on an industry-wide acceptable solution, is a mistake. It would require that any software tools coded between now and then to be redone. Instead, we focus on the first problem, which is establishing that there is a problem. We propose a nomenclature, but expect and look forward to many constructive criticisms to improve upon it. Meanwhile, this manuscript serves as a cite-able lexicon for anyone who has the need to describe these concepts (for example, in an algorithm manuscript) yet has no available way of doing so within page limits. The comparison of analyte MS1 signal is central to many proteomic (and other -omic) workflows. Standard vocabularies for mass spectrometry exist and provide good coverage for most experimental concepts, however their terms for data concepts and algorithms are either ambiguous or missing. Without a standard, unambiguous nomenclature, literature searches, algorithm reproducibility, and algorithm evaluation for MS-omics data processing are nearly impossible. We show how terms from current official ontologies are too vague or ambiguous to explicitly map molecular entities to MS signals, and we illustrate the inconsistency and ambiguity of current colloquially used terms. We propose a set of terms for MS1 data processing which consists of a limited number of base terms along with qualifier terms allowing a vast number of MS1 data concepts to be succinctly, precisely, and intuitively described. We suggest this nomenclature as a beginning to, not the culmination of, the standardization process.

#### You should be improving mzQuantML or mzIdentML instead of doing this

The mzQuantML and mzIdentML formats are not CVs. They are data formats (see previous objection).

## Conclusions

The ever-increasing number of MS-omics experiments drives a thriving MS-omics data processing algorithms field. However, the lack of an unambiguous vocabulary for MS-omics data concepts has created serious challenges for reproducibility and evaluation of data processing algorithms.

In this paper, we have highlighted the ambiguity of current vocabulary for MS-omics data processing. We propose an unambiguous vocabulary that spans the required granularity of MD data processing concepts together with a visual lexicon for the proposed terms. By adopting these terms, authors can facilitate reproduction of their algorithms succinctly by providing a crystal-clear set of meanings for terms they use, vastly improving the reproducibility of their work.

## Competing interests and declarations

The authors declare that they have no competing interests. The publication costs for this article were funded by the University of Montana Office of Research and Sponsored Programs.

This article has been published as part of *BMC Bioinformatics *Volume 16 Supplement 7, 2015: Selected articles from The 11th Annual Biotechnology and Bioinformatics Symposium (BIOT-2014): Bioinformatics. The full contents of the supplement are available online at http://www.biomedcentral.com/bmcbioinformatics/supplements/16/S7.

## Authors' contributions

RS, RMT and JTP all contributed to writing this manuscript.
